# Development of Paramyosin as a Vaccine Candidate for Schistosomiasis

**DOI:** 10.3389/fimmu.2015.00347

**Published:** 2015-07-21

**Authors:** Mario A. Jiz, Haiwei Wu, Remigio Olveda, Blanca Jarilla, Jonathan D. Kurtis

**Affiliations:** ^1^Department of Health, Research Institute for Tropical Medicine, Manila, Philippines; ^2^Center for International Health Research, Rhode Island Hospital, Brown University Medical School, Providence, RI, USA; ^3^Department of Pediatrics, Rhode Island Hospital, Brown University Medical School, Providence, RI, USA; ^4^Department of Pathology and Laboratory Medicine, Rhode Island Hospital, Brown University Medical School, Providence, RI, USA

**Keywords:** *Schistosoma japonicum*, vaccines, paramyosin, zoonoses, preclincal

## Abstract

Schistosomiasis, caused by three principal species of diecious trematodes (flatworms), currently afflicts over 250 million individuals, results in an estimated 2–15% chronic disability, and contributes to poor health and economic stagnation in endemic areas. Although schistosomiasis is effectively treated with praziquantel, rapid reinfection with rebound morbidity precludes effective control based on chemotherapy alone and justifies current efforts to develop vaccines for these parasites. Paramyosin (Pmy), an invertebrate muscle-associated protein, has emerged as a promising vaccine candidate for both *Schistosoma mansoni* and *Schistosoma japonicum*. Herein, we discuss the discovery of Pmy, its development as a vaccine candidate in rodents and bovines, as well as studies of naturally occurring immune responses to Pmy in prospective, observational human studies. We conclude with a proposed developmental plan to move Pmy toward Phase I clinical trials.

Schistosomiasis, caused by three principle species of diecious trematodes (flatworms), currently afflicts over 250 million people worldwide (1, 2) and results in 1.53 million DALYs lost per annum (3), although this is likely a considerable underestimate. A recent reassessment of the global burden of schistosomiasis suggests that the actual health burden is 4–30 times greater than the previous WHO estimate (1, 4). Infection is characterized by the presence of adult worms within the portal and mesenteric veins for *Schistosoma japonicum* and *Schistosoma mansoni* or within the veins draining the urinary bladder for *S. haematobium*. Chronic infection results in reduced childhood growth and nutritional status, anemia, hepatosplenomegaly, hepatic or urinary bladder fibrosis, and bleeding esophageal varices. End organ and systemic pathology result from granulomatous inflammation and fibrosis induced by parasite eggs trapped in host tissues.

Efforts to mitigate the impact of schistosomiasis in endemic regions have focused on chemotherapy of active infections with praziquantel (PZQ), control of intermediate snail hosts, and improved sanitation.

In longitudinal cohort studies in the Philippines, we have demonstrated that improvements in nutritional status and hemoglobin levels associated with PZQ treatment are transitory; both nutritional status and hemoglobin returned to their pre-treatment morbid levels within 9–12 months (5). In addition, “rebound” morbidity, in which repeated cycles of annual treatment and reinfection result in more aggressive morbidity, has been described (6). Therefore, annual treatment programs with PZQ are insufficient to reverse these morbidities, while resource requirements to shorten the treatment interval are prohibitive. Because current control strategies employing chemotherapy with PZQ have not reduced transmission and morbidity to acceptable levels, there is an urgent need for complementary approaches, such as vaccines for schistosomiasis control.

## Discovery of Paramyosin

In *S. mansoni*, Sher and colleagues demonstrated that mice vaccinated intradermally with schistosomula or adult worm extracts adjuvanted with BCG were protected from cercarial challenge (7). The sera from these mice were strikingly monospecific, recognizing a 97 kDa antigen in adult worm extracts and immunoprecipitating a 97 kDa antigen from detergent extracts of metabolically labeled worms (8). Size-fractionation analysis of adult worm extracts indicated that only fractions containing a protease sensitive 97 kDa antigen induced protection in this model (7). Monoclonal antibodies against Sm97 were developed from mice vaccinated intradermally with adult worm extract and BCG (8). Indirect immunofluorescence using monoclonal anti-Sm97 localized the antigen to regions just below the tegument and in the gut syncitia of adult worms. Sm97 was weakly recognized by chronically infected mouse sera and not recognized by sera from mice infected with irradiated cercariae (8). Immunoaffinity purified Sm97 was shown to elicit delayed-type hypersensitivity in intradermally vaccinated mice, suggesting that this molecule is also capable of evoking cell-mediated responses.

A rabbit anti-Sm97 serum immunoprecipitated a 97 kDa antigen from *in vitro* translation products of adult worm mRNA and identified a 1317 base pair clone, which encoded approximately 50% of the native protein (9). The clone had 36% homology with nematode myosin, but was designated paramyosin based on amino acid composition and cross reactivity of anti-Sm97 sera with other native paramyosins. Structural analysis of the deduced amino acid sequence and electron microscopy of purified schistosome paramyosin indicated that the protein adopts an alpha helical coiled-coil conformation with a seven residue repeat (10).

Paramyosin was localized to the contents of membrane bound elongate bodies within the tegument and subtegumental cell bodies using immunoelectron gold microscopy and polyclonal anti-paramyosin antisera (11). Paramyosin was in a non-filamentous form within these elongate bodies and staining was rarely observed in the thick filaments of cortical muscle.

A full length cDNA clone of *S. mansoni* paramyosin was sequenced and revealed significant homology to antigen B of *Taenia solium* and the IgG Fc γ-binding protein of *Taenia crassiceps* (12, 13). These homologous proteins bind the Fc region of IgG and collagen, and inhibit complement activation. Based on these homologies, schistosome paramyosin was shown to inhibit complement C1 and C9, and to bind polymeric collagen, and IgG (14–17). These immune-related properties may explain the tegumental localization of an otherwise muscle associated protein.

## Murine Protection Studies in *S. mansoni*

Passive transfer of monoclonal or polyclonal anti-paramyosin antibodies did not protect mice from cercarial challenge (18); however, immunization with 4 μg of native or 40 μg of a partial recombinant paramyosin fragment with BCG induced 39 and 26% protection, respectively. These data suggested that protection induced by paramyosin was cell mediated and not antibody dependent. Schistosome myosin and heterologous nematode paramyosin did not induce protection, suggesting the requirement of schistosome paramyosin specific epitopes for protection. In other experiments, 5 μg of native paramyosin induced 24–53% protection in mice without adjuvant (19).

Native and partial recombinant paramyosin stimulated IFN-γ production and macrophage killing of schistosomula in vaccinated mice. In addition, splenocytes from mice vaccinated with paramyosin/BCG were stimulated to produce IFN-γ by 3-h-old and 7-day-old schistosomula in a dose dependent fashion (18).

## Pmy Development in *S. japonicum*

In contradistinction to the *S. mansoni* model, a mouse monoclonal IgE antibody, subsequently demonstrated to recognize paramyosin (20), was shown to confer 19–58% protection against *S. japonicum* cercarial challenge following passive transfer (21, 22). In addition, the monoclonal stimulated eosinophill-mediated killing of schistosomula *in vitro* (22).

To determine if paramyosin would also induce protection against *S. japonicum*, we biochemically purified paramyosin from *S. japonicum* adult worms. SDS-PAGE demonstrated a single protein with a molecular weight of 97 kDa. In four separate experiments, vaccination of mice with *S. japonicum* paramyosin without adjuvant induced significant resistance (62–86%, *p* < 0.001) against cercarial challenge as compared to controls. These data suggest that *S. japonicum* paramyosin represents a promising candidate vaccine (23). Paramyosin has also been evaluated as a component of a multi-antigen DNA-based vaccine in mice; however, delivery in this format has not augmented the level of protection seen with recombinant or biochemically purified native paramyosin (24).

Based on these encouraging protection data, McManus and colleagues cloned the full length paramyosin gene from an *S. japonicum* cDNA library probed with a hyperimmune rabbit sera (25), while Nara et al. independently isolated the full-length cDNA of Sj97 by screening a distinct library using a mouse monoclonal IgE antibody, which recognizes a 97 kDa surface molecule on *S. japonicum* larvae (26). Alignment of the predicted amino acid sequences of *S. mansoni* and *S. japonicum* paramyosins revealed 95% identity.

## Sj97 is the Target of Protective Th2 Biased Cytokine Responses in Humans

We conducted a longitudinal treatment–reinfection study design with 616 *S. japonicum* infected participants, 7–30 years of age. We evaluated the relationship between cytokine responses made by PMBCs in response to *S. japonicum* soluble adult worm extract (SWAP), Sj97, Sj67, and Sj22.6, measured 4 weeks after treatment with PZQ, and resistance to reinfection in a population from Leyte, The Philippines. Reinfection was measured every 3 months for a total of 18 months by duplicate Kato–Katz assessment on three stools per person per timepoint. We employed repeated measures models adjusted for both repeated measures within person, clustering by household and several potential confounders of reinfection intensity including: directly observed water contact, socio-economic status, age, sex, village, and baseline intensity of infection.

**Figure 1:**
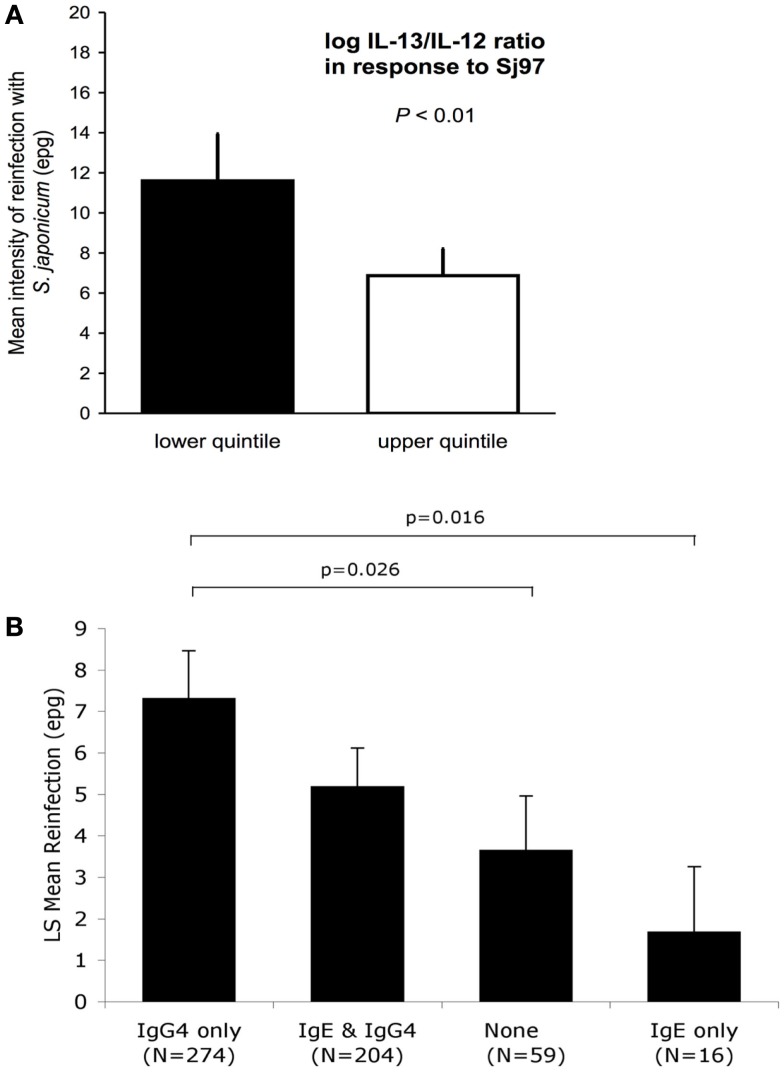
**(A)** Intensity of reinfection with *S. japonicum* 18 months after treatment with PZQ, as predicted by cytokine ratios in responses to SWAP (*N* = 493). White and black bars represent the back-transformed LS mean intensity of reinfection for the upper and lower quintile of IL13/IL12 log cytokine-ratio distribution. Error bars represent SEs. LS mean estimates, SEs, and *p*-values for difference in means between quintiles are adjusted for confounders and clustering. Adapted from Ref. (26). **(B)** IgE responses to rSj97 (paramyosin) predict resistance to *S. japonicum* reinfection at 12 months post-treatment, and are attenuated by lgG_4_. Least square (LS) means represent the mean reinfection egg burden after adjusting for potential confounders and clustering by household in a repeated measures model using the combined Sj97 IgE and lgG_4_ response variable (*p* = 0.023 for time by combined lgE–lgG_4_ variable interaction). Confounders in this model include age, gender, village of residence, exposure, and baseline intensity. *p*-values are for detailed comparisons between “lgG_4_ only” and the rest of the groups. Error bars represent SEs. Reprinted from Ref. (27).

*Schistosoma japonicum* transmission was high: 54.8 and 91.1% were reinfected within 6 and 18 months, respectively. A Th2 bias in the following cytokine ratios, IL-5/IL-12, IL-13/IL-12, IL-4/IFN-g, IL-5/IFN-γ, and IL-13/IFN-γ, in response to Sj97, predicted a 30–41% lower intensity of reinfection (all *p* < 0.05) after adjustment for potential confounders. An example of one of these protective relationships (for IL-13/IFN-γ ratio) is presented in Figure 1A (27). Similar results were found for responses to SWAP.

## Sj97 is the Target of Protective IgE Responses in Humans

Using the same study population and analytic approach described for the cytokine analyses, we have evaluated the relationship between antibodies to rSj97 and resistance to reinfection in humans. We obtained serum 4 weeks post-treatment and measured anti-rSj97 IgE, IgA, and IgG subclass specific antibody levels using a bead-based assay. Responders were defined based on the mean +2 SD of the fluorescence values obtained in a group of 10 unexposed North American controls. In repeated measures models, individuals with IgE but not IgG4 responses to rSj97 had a 77% lower intensity of reinfection at 12 months compared to individuals with IgG4 but not IgE responses, even after adjusting for potential confounders including directly observed water contact, village, age, sex, and baseline intensity of infection (*p* = 0.016) (28), see Figure 1B.

## Bovine Vaccine Trials

Using partially purified rSj97, McManus et al. (29) demonstrated that Chinese buffalo immunized with rSj97 in Quill A developed specific anti-Sj97 antibodies and had 34% fewer worms after laboratory challenge compared to controls. Importantly, the authors also acknowledged that, “*whereas the yields of rec-Sj97 are sufficient for small trials, the expression levels are very low and are inadequate for large-scale use*.”

## Pilot Scale Expression, Purification, and Lyophilization of rSj97

As indicated above, recombinant Sj97 has not been successfully produced at pilot scale despite considerable efforts (29, 30). After discovering that Th2 and IgE responses to Sj97 were associated with marked resistance to reinfection in *humans*, we have focused our efforts on overcoming this scale-up challenge (31). Our typical yield from a single 10 L fermentation is 600 lyophilized vials at >0.250 mg rSj97/vial.

## Characterization of rSj97

**Figure 2:**
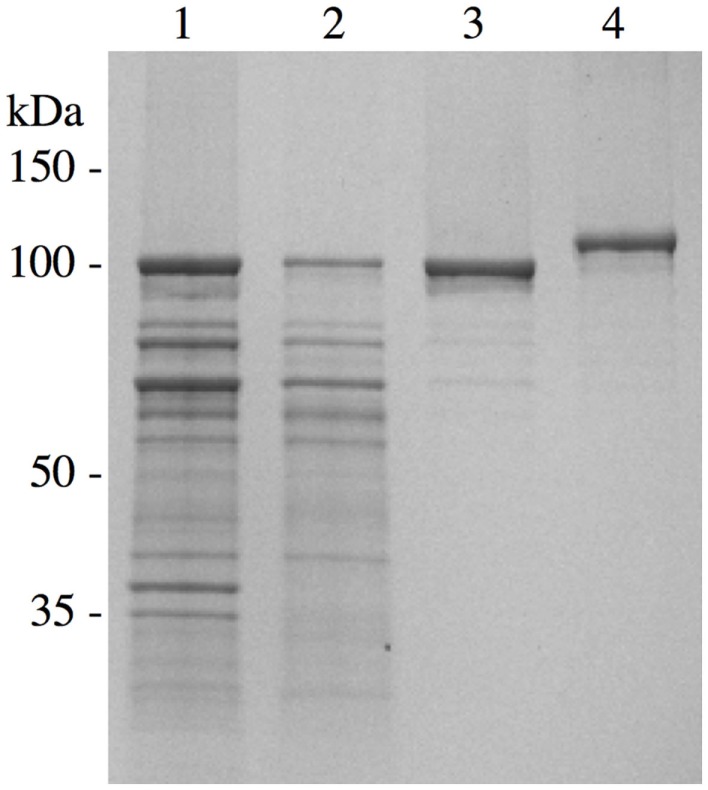
**Chromatographic purification of *S. japonicum* paramyosin**. A pET-30 plasmid containing paramyosin was expressed and purified similarly as described (28). Lane 1, inclusion body preparation; lane 2, anion exchange chromatography; lane 3, size exclusion chromatography; lane 4, purified *S*. *japonicum* paramyosin with a thioredoxin fusion tag. Reprinted from Ref. (30).

We have conducted extensive functional, biochemical, biophysical, and immunologic analyses on rSj97 to demonstrate that our recombinant protein is free of concerning contaminants, is correctly folded, and has appropriate functional properties. Electrophoretic mobility and purity (Figure 2), sterility, endotoxin level (by FDA approved test), residual SDS concentration, identity (LC-MS), secondary and tertiary structural analysis (CD), collagen binding, IgG binding, and stability (>12 months) gave expected results (31).

## Future Product Development for rSj97

Together with colleagues, we are actively pursuing vaccination trials in both murine and bovine models with encouraging preliminary results. These experiments are designed to evaluate several doses of antigen as well as several adjuvants suitable for both veterinary and human use. Because *Schistosomiasis japonica* is a zoonosis, with bovines playing a key role in transmission to humans (32, 33), we are also evaluating the impact of bovine vaccination on human incidence of infection in endemic communities. Sj97 is a target of protective human IgE responses; therefore, we are also evaluating the potential for inducing hypersensitivity reactions by skin testing with rSj97 in infected bovines, again with encouraging preliminary results. If successful, these data, together with our GMP ready process for expression and purification, will form a compelling argument to move recombinant paramyosins toward Phase I trials in humans.

## Conflict of Interest Statement

The authors declare that the research was conducted in the absence of any commercial or financial relationships that could be construed as a potential conflict of interest.
